# 1,2-Bis(1*H*-tetra­zol-5-yl)benzene dihydrate

**DOI:** 10.1107/S1600536809018224

**Published:** 2009-05-20

**Authors:** Hai-Jun Xu, Yi-Jie Pan, Li-Jing Cui

**Affiliations:** a Ordered Matter Science Research Center, College of Chemistry and Chemical Engineering, Southeast University, Nanjing 210096, People’s Republic of China

## Abstract

The asymmetric unit of the title compound, C_8_H_6_N_8_·2H_2_O, contains one half-mol­ecule, with the benzene ring on a centre of symmetry, and two uncoordinated water mol­ecules. The benzene ring is oriented at a dihedral angle of 34.43 (12)° with respect to the tetra­zole ring. Strong O—H⋯N hydrogen bonds link the water mol­ecules to the N atoms of the tetra­zole ring. In the crystal structure, strong inter­molecular O—H⋯O and O—H⋯N hydrogen bonds link the mol­ecules into a network. One of the water H atoms is disordered over two positions and was refined with occupancies of 0.50.

## Related literature

For general background, see: Luo *et al.* (2006 ▶). For related structures, see: Guzei & Bikzhanova (2002 ▶); Pan *et al.* (2007 ▶).
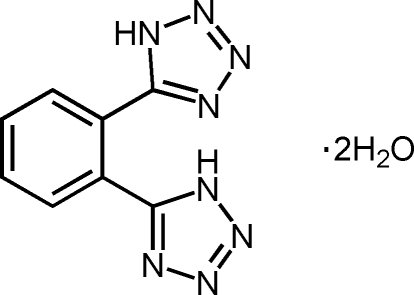


## Experimental

### 

#### Crystal data

C_8_H_6_N_8_·2H_2_O
*M* *_r_* = 286.27
Monoclinic, 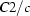

*a* = 14.510 (3) Å
*b* = 12.427 (3) Å
*c* = 7.2576 (15) Å
β = 96.29 (3)°
*V* = 1300.7 (5) Å^3^
*Z* = 4
Mo *K*α radiation
μ = 0.12 mm^−1^
*T* = 294 K
0.20 × 0.20 × 0.20 mm

#### Data collection

Rigaku SCXmini diffractometer
Absorption correction: multi-scan (Blessing, 1995 ▶) *T*_min_ = 0.971, *T*_max_ = 0.979
5963 measured reflections
1276 independent reflections
1041 reflections with *I* > 2σ(*I*)
*R*_int_ = 0.044

#### Refinement

*R*[*F* ^2^ > 2σ(*F* ^2^)] = 0.040
*wR*(*F* ^2^) = 0.098
*S* = 1.06
1276 reflections
101 parameters
H atoms treated by a mixture of independent and constrained refinement
Δρ_max_ = 0.20 e Å^−3^
Δρ_min_ = −0.15 e Å^−3^

### 

Data collection: *CrystalClear* (Rigaku/MSC (2005 ▶); cell refinement: *CrystalClear*; data reduction: *CrystalClear*; program(s) used to solve structure: *SHELXS97* (Sheldrick, 2008 ▶); program(s) used to refine structure: *SHELXL97* (Sheldrick, 2008 ▶); molecular graphics: *ORTEP-3 for Windows* (Farrugia, 1997 ▶); software used to prepare material for publication: *SHELXL97*.

## Supplementary Material

Crystal structure: contains datablocks I, global. DOI: 10.1107/S1600536809018224/hk2687sup1.cif

Structure factors: contains datablocks I. DOI: 10.1107/S1600536809018224/hk2687Isup2.hkl

Additional supplementary materials:  crystallographic information; 3D view; checkCIF report

## Figures and Tables

**Table 1:** Hydrogen-bond geometry (Å, °)

*D*—H⋯*A*	*D*—H	H⋯*A*	*D*⋯*A*	*D*—H⋯*A*
N4—H4*A*⋯O1*W*	0.91 (2)	1.78 (2)	2.682 (2)	171.9 (19)
O1*W*—H1*WA*⋯N2^i^	0.85	2.02	2.8658 (19)	173
O1*W*—H1*WB*⋯O2*W*^ii^	0.85	1.98	2.813 (2)	168
O2*W*—H2*WA*⋯N1	0.85	2.06	2.896 (2)	169
O2*W*—H2*WB*⋯O2*W*^iii^	0.85	1.97	2.813 (3)	170
O2*W*—H2*WC*⋯O2*W*^iv^	0.85	2.01	2.814 (3)	158

Symmetry codes: (i) 
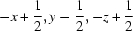
; (ii) 
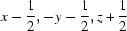
; (iii) 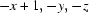; (iv) 
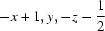
.

**Fig. 1.**
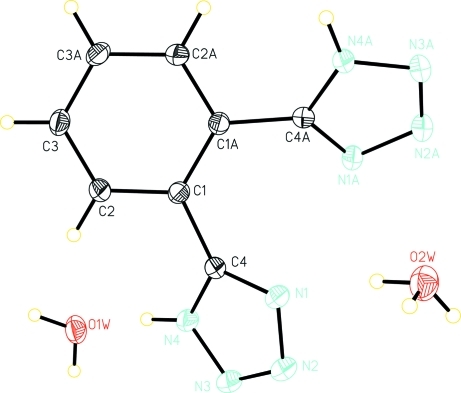
The molecular structure of the title molecule, with the atom-numbering scheme. Displacement ellipsoids are drawn at the 30% probability level.

**Fig. 2.**
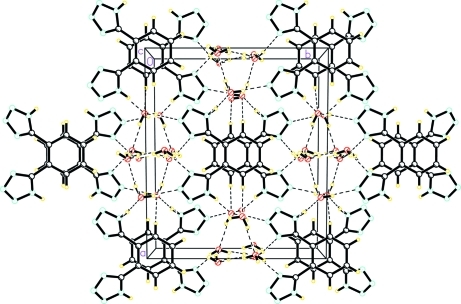
A partial packing diagram of the title compound. Hydrogen bonds are shown as dashed lines.

## supplementary crystallographic information

### Comment

The tetrazole functional group has currently been received considerable attention mainly because of a wide range of applications in coordination chemistry, medicinal chemistry and materials science (Luo *et al.*, 2006). However, there are a few crystal structure reports of organic tetrazolates compounds in the literature (Guzei & Bikzhanova, 2002). We reported herein the synthesis and the crystal structure of the title compound.

The asymmetric unit of the title compound contains one-half molecule, with benzene ring on a centre of symmetry, and two uncoordinated water molecules (Fig. 1). The bond lengths and angles are in accordance with the corresponding values reported (Pan *et al.*, 2007). The benzene ring is oriented with respect to the tetrazole ring at a dihedral angle of 34.43 (12)°. Strong intramolecular O-H···N hydrogen bonds (Table 1) link the water molecules to the nitrogens of the tetrazole ring.

In the crystal structure, strong intermolecular O-H···O and O-H···N hydrogen bonds (Table 1) link the molecules into a network (Fig. 2), in which they may be effective in the stabilization of the structure.

### Experimental

For the preparation of the title compound, phthalonitrile (1.28 g, 10 mmol), NH_4_Cl (1.38 g, 26 mmol) and NaN_3_ (1.69 g, 13 mmol) were dissolved in DMF (60 ml). The mixture was heated to 353 K, and stirred for 48 h. Then, it was cooled to room temperature and poured into cold water and acidified to pH = 2 with concentrated hydrochloric acid. After 12 h at 277 K, the suspension was filtrated, and the residue was washed with H_2_O and H_2_O/EtOH (1/1), and then dried. Crystals suitable for X-ray analysis were obtained from an EtOH solution.

### Refinement

One of the H atoms bonded to O2W was disordered. During the refinement process, the disordered H atom was refined with occupancies of 0.50 and 0.50. H atom (for NH) was located in difference Fourier synthesis and refined isotropically. The remaining H atoms were positioned geometrically with O-H = 0.85 Å (for H_2_O) [U_iso_(H) = 0.060 (15)-0.086 (9) Å^2^] and C-H = 0.93 Å, for aromatic H atoms and constrained to ride on their parent atoms, with U_iso_(H) = 1.2U_eq_(C).

### Crystal data

**Table:**

C_8_H_6_N_8_·2H_2_O	*F*(000) = 600
*M**_r_* = 286.27	*D*_x_ = 1.462 Mg m^−^^3^
Monoclinic, *C*2/*c*	Mo *K*α radiation, λ = 0.71073 Å
Hall symbol: -C 2yc	Cell parameters from 5656 reflections
*a* = 14.510 (3) Å	θ = 3.3–27.5°
*b* = 12.427 (3) Å	µ = 0.12 mm^−^^1^
*c* = 7.2576 (15) Å	*T* = 294 K
β = 96.29 (3)°	Prism, colorless
*V* = 1300.7 (5) Å^3^	0.20 × 0.20 × 0.20 mm
*Z* = 4	

### Data collection

**Table:**

Rigaku SCXmini diffractometer	1276 independent reflections
Radiation source: fine-focus sealed tube	1041 reflections with *I* > 2σ(*I*)
graphite	*R*_int_ = 0.044
Detector resolution: 13.6612 pixels mm^-1^	θ_max_ = 26.0°, θ_min_ = 3.3°
ω scans	*h* = −17→17
Absorption correction: multi-scan (Blessing, 1995)	*k* = −15→15
*T*_min_ = 0.971, *T*_max_ = 0.979	*l* = −8→8
5963 measured reflections	

### Refinement

**Table:**

Refinement on *F*^2^	Secondary atom site location: difference Fourier map
Least-squares matrix: full	Hydrogen site location: inferred from neighbouring sites
*R*[*F*^2^ > 2σ(*F*^2^)] = 0.040	H atoms treated by a mixture of independent and constrained refinement
*wR*(*F*^2^) = 0.098	*w* = 1/[σ^2^(*F*_o_^2^) + (0.0394*P*)^2^ + 0.846*P*] where *P* = (*F*_o_^2^ + 2*F*_c_^2^)/3
*S* = 1.06	(Δ/σ)_max_ < 0.001
1276 reflections	Δρ_max_ = 0.20 e Å^−^^3^
101 parameters	Δρ_min_ = −0.15 e Å^−^^3^
0 restraints	Extinction correction: *SHELXL97* (Sheldrick, 2008), Fc^*^=kFc[1+0.001xFc^2^λ^3^/sin(2θ)]^-1/4^
Primary atom site location: structure-invariant direct methods	Extinction coefficient: 0.0220 (19)

### Special details

**Table:**

Geometry. All e.s.d.'s (except the e.s.d. in the dihedral angle between two l.s. planes) are estimated using the full covariance matrix. The cell e.s.d.'s are taken into account individually in the estimation of e.s.d.'s in distances, angles and torsion angles; correlations between e.s.d.'s in cell parameters are only used when they are defined by crystal symmetry. An approximate (isotropic) treatment of cell e.s.d.'s is used for estimating e.s.d.'s involving l.s. planes.
Refinement. Refinement of *F*^2^ against ALL reflections. The weighted *R*-factor *wR* and goodness of fit *S* are based on *F*^2^, conventional *R*-factors *R* are based on *F*, with *F* set to zero for negative *F*^2^. The threshold expression of *F*^2^ > σ(*F*^2^) is used only for calculating *R*-factors(gt) *etc*. and is not relevant to the choice of reflections for refinement. *R*-factors based on *F*^2^ are statistically about twice as large as those based on *F*, and *R*- factors based on ALL data will be even larger.

### Fractional atomic coordinates and isotropic or equivalent isotropic displacement parameters (Å^2^)

**Table:**

	*x*	*y*	*z*	*U*_iso_*/*U*_eq_	Occ. (<1)
O1W	0.20523 (9)	−0.46870 (10)	0.4630 (2)	0.0479 (4)	
H1WA	0.1982	−0.5343	0.4316	0.086 (9)*	
H1WB	0.1528	−0.4390	0.4684	0.084 (9)*	
O2W	0.52104 (9)	−0.10467 (11)	−0.0561 (2)	0.0467 (4)	
H2WA	0.4931	−0.1528	−0.0003	0.076 (8)*	
H2WB	0.5036	−0.0452	−0.0135	0.060 (15)*	0.50
H2WC	0.4968	−0.1163	−0.1663	0.078 (19)*	0.50
N1	0.40777 (10)	−0.24613 (11)	0.1426 (2)	0.0332 (4)	
N2	0.33249 (10)	−0.18460 (11)	0.1621 (2)	0.0397 (4)	
N3	0.27393 (10)	−0.23572 (12)	0.2522 (2)	0.0417 (4)	
N4	0.31147 (10)	−0.33246 (12)	0.2923 (2)	0.0353 (4)	
H4A	0.2797 (14)	−0.3834 (17)	0.350 (3)	0.053 (6)*	
C1	0.45124 (10)	−0.43665 (12)	0.2407 (2)	0.0264 (4)	
C2	0.40490 (11)	−0.53513 (12)	0.2324 (2)	0.0308 (4)	
H2A	0.3404	−0.5358	0.2205	0.037*	
C3	0.45222 (11)	−0.63163 (13)	0.2414 (2)	0.0327 (4)	
H3A	0.4198	−0.6963	0.2360	0.039*	
C4	0.39372 (11)	−0.33838 (12)	0.2258 (2)	0.0277 (4)	

### Atomic displacement parameters (Å^2^)

**Table:**

	*U*^11^	*U*^22^	*U*^33^	*U*^12^	*U*^13^	*U*^23^
O1W	0.0353 (8)	0.0370 (8)	0.0737 (10)	−0.0091 (6)	0.0168 (7)	−0.0132 (7)
O2W	0.0504 (9)	0.0396 (8)	0.0527 (9)	−0.0046 (6)	0.0166 (7)	0.0005 (7)
N1	0.0323 (8)	0.0253 (7)	0.0421 (9)	0.0035 (6)	0.0052 (6)	0.0021 (6)
N2	0.0379 (9)	0.0283 (8)	0.0528 (10)	0.0071 (6)	0.0040 (7)	0.0015 (7)
N3	0.0321 (9)	0.0304 (8)	0.0630 (11)	0.0082 (6)	0.0067 (8)	−0.0009 (7)
N4	0.0271 (8)	0.0262 (7)	0.0537 (10)	0.0020 (6)	0.0103 (7)	0.0017 (7)
C1	0.0265 (8)	0.0235 (8)	0.0298 (9)	0.0009 (6)	0.0059 (6)	0.0010 (7)
C2	0.0250 (9)	0.0280 (9)	0.0401 (10)	−0.0024 (6)	0.0065 (7)	−0.0014 (7)
C3	0.0357 (9)	0.0225 (8)	0.0408 (10)	−0.0050 (7)	0.0082 (8)	−0.0005 (7)
C4	0.0235 (8)	0.0255 (8)	0.0341 (9)	−0.0011 (6)	0.0026 (7)	−0.0017 (7)

### Geometric parameters (Å, °)

**Table:**

O1W—H1WA	0.8500	C1—C2	1.394 (2)
O1W—H1WB	0.8501	C1—C1^i^	1.407 (3)
O2W—H2WA	0.8499	C1—C4	1.476 (2)
O2W—H2WB	0.8500	C2—C3	1.380 (2)
O2W—H2WC	0.8500	C2—H2A	0.9300
N1—N2	1.353 (2)	C3—C3^i^	1.378 (3)
N2—N3	1.294 (2)	C3—H3A	0.9300
N3—N4	1.339 (2)	C4—N1	1.322 (2)
N4—H4A	0.91 (2)	C4—N4	1.338 (2)
			
H1WA—O1W—H1WB	110.3	C2—C1—C4	117.17 (14)
H2WA—O2W—H2WB	105.2	C1^i^—C1—C4	124.17 (8)
H2WA—O2W—H2WC	99.2	C3—C2—C1	121.71 (15)
H2WB—O2W—H2WC	112.4	C3—C2—H2A	119.1
C4—N1—N2	105.99 (13)	C1—C2—H2A	119.1
N3—N2—N1	110.96 (14)	C3^i^—C3—C2	119.65 (9)
N2—N3—N4	106.06 (13)	C3^i^—C3—H3A	120.2
C4—N4—N3	109.19 (14)	C2—C3—H3A	120.2
C4—N4—H4A	130.1 (13)	N1—C4—N4	107.80 (14)
N3—N4—H4A	120.6 (13)	N1—C4—C1	129.54 (15)
C2—C1—C1^i^	118.65 (9)	N4—C4—C1	122.58 (14)

Symmetry codes: (i) −*x*+1, *y*, −*z*+1/2.

### Hydrogen-bond geometry (Å, °)

**Table:**

*D*—H···*A*	*D*—H	H···*A*	*D*···*A*	*D*—H···*A*
N4—H4A···O1W	0.91 (2)	1.78 (2)	2.682 (2)	171.9 (19)
O1W—H1WA···N2^ii^	0.85	2.02	2.8658 (19)	173
O1W—H1WB···O2W^iii^	0.85	1.98	2.813 (2)	168
O2W—H2WA···N1	0.85	2.06	2.896 (2)	169
O2W—H2WB···O2W^iv^	0.85	1.97	2.813 (3)	170
O2W—H2WC···O2W^v^	0.85	2.01	2.814 (3)	158

Symmetry codes: (ii) −*x*+1/2, *y*−1/2, −*z*+1/2; (iii) *x*−1/2, −*y*−1/2, *z*+1/2; (iv) −*x*+1, −*y*, −*z*; (v) −*x*+1, *y*, −*z*−1/2.
